# The Development and Pilot Clinical Study of CD147 Targeted Antagonistic Peptide Probe for Tumor Imaging

**DOI:** 10.1002/advs.202522550

**Published:** 2026-05-26

**Authors:** Xiaokun Ma, Rui Guo, Dongfeng Niu, Yufei Song, Zhenyao Zhang, Xiangxi Meng, Lili Mao, Haifeng Huang, Hua Zhu, Zhi Yang, Teli Liu

**Affiliations:** ^1^ State Key Laboratory of Holistic Integrative Management of Gastrointestinal Cancers, Beijing Key Laboratory of Research, Investigation and Evaluation of Radiopharmaceuticals, NMPA Key Laboratory For Research and Evaluation of Radiopharmaceuticals (National Medical Products Administration), Department of Nuclear Medicine Peking University Cancer Hospital & Institute Beijing China; ^2^ Beijing Key Laboratory of Research, Department of Pathology, Key Laboratory of Carcinogenesis and Translational Research (Ministry of Education) Peking University Cancer Hospital & Institute Beijing China; ^3^ Department of Genitourinary Oncology, Key Laboratory of Carcinogenesis and Translational Research (Ministry of Education) Peking University Cancer Hospital & Institute Beijing China; ^4^ Department of Orthopeadics Guizhou Provincial People's Hospital Guiyang China

**Keywords:** [^68^Ga]Ga, antagonistic peptide, CD147, malignant tumors, PET/CT imaging

## Abstract

Extracellular matrix metalloproteinase inducer (EMMPRIN/CD147) is highly expressed in multiple malignancies and represents a potential therapeutic target. Monitoring CD147 expression noninvasively may facilitate tumor detection and guide therapeutic strategies. We developed a novel peptide‐based PET probe, [^68^Ga]Ga‐DOTA‐AP9, and validated its pharmacokinetics and specificity in preclinical and translational uEXPLORER total‐body PET/CT studies. In preclinical models, [^68^Ga]Ga‐DOTA‐AP9 showed high binding affinity and specific uptake in CD147‐positive A375 tumors. Encouraged by the favorable tumor‐to‐background contrast and safety profile observed in animals, [^68^Ga]Ga‐DOTA‐AP9 total‐body PET/CT imaging was performed in eleven participants with diverse cancer types and varying CD147 expression levels. Among the eleven participants, tumor lesions showed a maximum SUV_max_ of 10.1 at 30 min p.i., and uptake correlated strongly with CD147 (r  =  0.7981, *P* < 0.001), MCT1 (r  =  0.8555, *P* < 0.001), and Ki67 (r  =  0.8036, *P* < 0.001), but not GLUT1 (*P*  =  0.0668). Notably, [^68^Ga]Ga‐DOTA‐AP9 uptake more accurately reflected CD147 expression than [^18^F]F‐FDG, with divergent uptake patterns in some lesions. These findings support that [^68^Ga]Ga‐DOTA‐AP9 enables sensitive, noninvasive detection and quantitative assessment of CD147‐positive tumors in vivo, provides complementary diagnostic information to [^18^F]F‐FDG PET, and holds significant potential for patient stratification, treatment selection, therapeutic response monitoring, and guiding precision oncology strategies.

## Graphical abstract

This study establishes [^68^Ga]Ga‐DOTA‐AP9 as a first‐in‐human CD147‐targeted PET tracer with favorable safety and specific tumor uptake. Tracer accumulation correlates with CD147 expression in patients, enabling noninvasive quantification of CD147‐positive malignancies. These findings support a promising theranostic strategy for patient stratification in CD147‐targeted therapy and for advancing understanding of CD147 biology in human cancers and clinical translation in precision oncology.

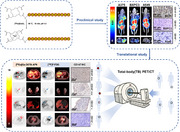

## 1 Introduction

Cancer remains a leading cause of mortality worldwide, imposing an increasing public health burden across diverse populations. Despite substantial advances in cancer therapy, early diagnosis and timely intervention remain pivotal for reducing cancer‐related morbidity and mortality [1, 2]. However, metastasis and recurrence continue to be the principal drivers of therapeutic failure and poor long‐term survival [3]. Accordingly, the development of precise diagnostic tools targeting the key molecular mediators underlying these processes is critical for improving clinical outcomes [4, 5].

Extracellular matrix metalloproteinase inducer (EMMPRIN), also known as CD147 or Basigin, is a transmembrane glycoprotein belonging to the immunoglobulin superfamily. CD147 is broadly expressed in both hematopoietic and non‐hematopoietic cells, including monocytes, granulocytes, epithelial, and endothelial cells. Importantly, CD147 is overexpressed in a wide range of aggressive malignancies, such as colorectal cancer, breast cancer, hypopharyngeal squamous cell carcinoma, hepatocellular carcinoma, and malignant melanoma, among others [6, 7, 8], and has emerged as a central regulator of tumor progression. Functionally, CD147 facilitates invasion and metastasis by inducing extracellular matrix metalloproteinases (MMPs) [9, 10, 11], and vascular endothelial growth factor (VEGF) [12], and also promotes tumor survival and metabolic adaptation through its chaperone function for monocarboxylate transporters (MCTs). These multifaceted oncogenic roles have established CD147 as a compelling target for cancer diagnosis and targeted therapy.

The clinical relevance of CD147 is underscored by emerging targeted therapeutic strategies [13]. The radioiodinated F(ab')_2_ fragment Metuximab ([^131^I]I‐Metuximab, Licartin) has been approved in China for hepatocellular carcinoma (HCC) treatment as a radioimmunotherapeutic agent, either alone or in combination with radiofrequency ablation (RFA) or transcatheter arterial chemoembolization (TACE) [14, 15, 16]. Additionally, CD147‐directed CAR‐T and CAR‐NK cell therapies have been developed, with several registered clinical trials having been initiated for the treatment of HCC (NCT03993743, ChiCTR2100045721, ChiCTR2000041023), non‐Hodgkin's lymphoma (NCT05013372), and glioma (NCT04045847) [17, 18].

However, a major barrier to the advancement of these CD147‐targeted therapies is the lack of robust, noninvasive tools for quantitatively assessing CD147 expression in vivo. Such imaging tools are crucial for patient stratification, target validation, and monitoring therapeutic response. Aya et al. developed an [^89^Zr]Zr labeled anti‐CD147 antibody ([^89^Zr]Zr‐059‐093) for imaging CD147‐positive pancreatic tumors, highlighting its potential for patient stratification in CD147‐targeted therapies [19]. Jiajun et al. reported an iodine‐131‐labeled Meplazumab ([^131^I]I‐Meplazumab), and assessed its biodistribution and dosimetry in healthy volunteers to explore its therapeutic potential for Coronavirus disease 2019 (COVID‐19) [20]. Our group has also explored radioiodine‐labeled anti‐CD147 agents ([^124/125^I]I‐anti‐CD147 and [^124^I]I‐NB147) [7, 21]. Although these imaging agents showed promising preclinical imaging performance in multiple cancers (e.g., malignant melanoma, pancreatic cancer, and triple‐negative breast cancer), with tumor uptake correlating with CD147 expression levels. However, their clinical translation has been limited by suboptimal pharmacokinetics and regulatory challenges associated with radioiodinated probes.

Peptide‐based PET radiotracers offer a promising alternative, combining high target specificity with favorable pharmacokinetics, including rapid tumor targeting and early lesion detection, thereby facilitating timely and accurate diagnosis [22]. Moreover, compared with small‐molecule ligands, peptide ligands provide greater functional versatility, allowing facile conjugation with diverse chelators, radioisotopes, and radionuclides tailored to specific biological targets [23]. Their ability to form multipoint interactions with target proteins further enhances binding specificity and expands the range of tractable molecular targets for precision imaging [24].

AP9, a CD147 antagonist peptide, was first identified by Chen's group in 2003 through screening of a 12‐mer phage‐displayed random peptide library [25]. It exhibited high inhibitory rates against hepatoma cell invasion (81.2%) and chorioallantoic membrane angiogenesis (77.8%), and was further shown to interfere with HCC cell adhesion to collagen IV, laminin, and fibroblasts [26, 27]. To address the unmet need for clinical CD147 imaging, we developed [^68^Ga]Ga‐DOTA‐AP9, a novel gallium‐68‐labeled peptide probe. This was achieved by introducing an alanine residue and conjugating DOTA to the N‐terminus of AP9, followed by radiolabeling with [^68^Ga]GaCl_3_.

In this study, we report the preclinical evaluation and first‐in‐human application of [^68^Ga]Ga‐DOTA‐AP9. We demonstrate its specific targeting of CD147‐positive tumors, favorable safety characteristics, and a significant association between tracer uptake and CD147 expression levels in patients. Collectively, this work establishes a promising theranostic platform for the noninvasive quantification of CD147, with immediate translational potential for guiding patient selection in CD147‐targeted therapies and for deepening our understanding of the biological role of CD147 in human malignancies.

## 2 Results

### 2.1 Synthesis and Molecular Docking Analysis

The DOTA‐conjugated precursor, DOTA‐AP9, was synthesized via solid‐phase peptide synthesis (Figure S1) and purified by high‐performance liquid chromatography (HPLC), achieving a purity of 96% (Figure S2A). The molecular identity was confirmed by ESI‐MS, which displayed a peak at m/z 667.24 corresponding to the [M+3H]^3^^+^ ion, consistent with the calculated molecular weight of 1999.23 Da for DOTA‐AP9 (Figure S2B).

**FIGURE 1:**
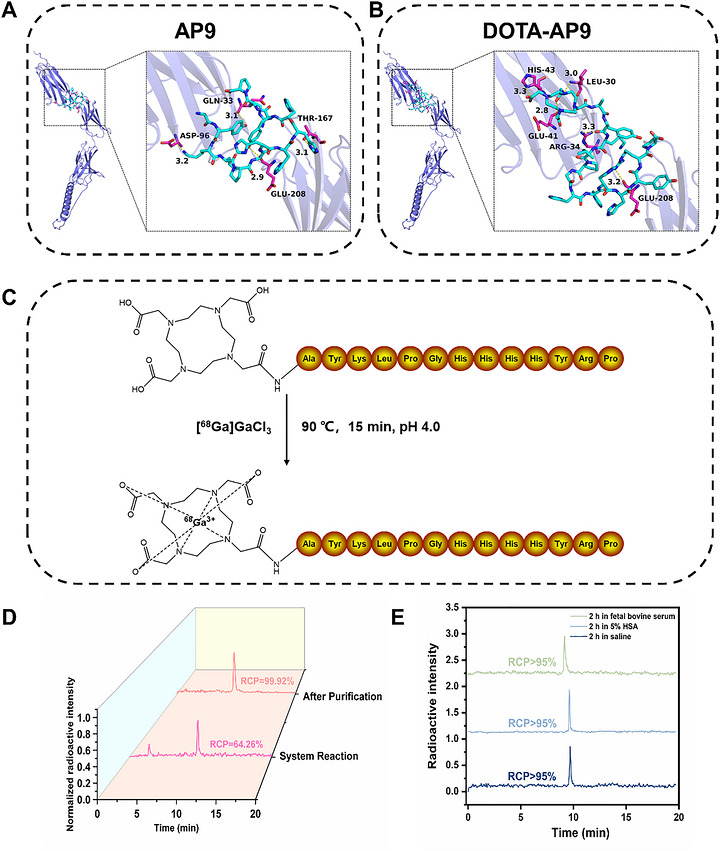
**Molecular docking analysis and radiolabeling of [^68^Ga]Ga‐DOTA‐AP9**. (A, B) Molecular docking of AP9 and DOTA‐AP9 to human CD147 protein (Purple: CD147 protein; Blue: AP9 or DOTA‐AP9; Magentas stick structures: binding sites; Yellow dashed lines: hydrogen bonds; Green dashed lines: hydrophobic interactions); (C) Radiolabeling scheme of [^68^Ga]Ga‐DOTA‐AP9; (D) Radio‐HPLC chromatograms of [^68^Ga]Ga‐DOTA‐AP9 before and after purification; (E) Radio‐HPLC chromatograms of [^68^Ga]Ga‐DOTA‐AP9 incubated in saline, 5% HAS, and FBS for 2 h.

Molecular docking simulations using AlphaFold 3.0 were performed to predict the binding conformations of both AP9 and DOTA‐AP9 with CD147. Analysis of the top‐ranked binding poses revealed distinct interaction profiles. As shown in Figure 1A, B, AP9 formed hydrogen bonds with CD147 residues GLU208, GLN33, THR167, and ASP96, and exhibited a binding energy of –4.9 kcal/mol. In contrast, DOTA‐AP9 engaged in additional hydrogen bonds with GLU208, GLU41, HIS43, ARG34, and LEU30, and demonstrated a stronger binding energy of −5.6 kcal/mol. These computational results suggest that conjugation with DOTA enhances the binding affinity of the AP9 peptide to CD147, potentially improving its targeting efficacy.

### 2.2 Radiolabeling and Physicochemical Properties Investigation of [^68^Ga]Ga‐DOTA‐AP9

Radiolabeling of DOTA‐AP9 with Gallium‐68 was carried out efficiently, yielding a labeling efficiency of 55.77 ± 6.65% (n = 3, Figure 1C). Following purification using a C18 Light Cartridge, the radiochemical purity exceeded 99%, as determined by radio‐HPLC (retention time = 9.17 min, Figure 1D). The specific activity of the final product ranged from 12.9 to 34.6 GBq/µmol. Comprehensive quality control data are summarized in Table 1.

**TABLE 1:** Quality Control of [^68^Ga]Ga‐ DOTA‐AP9.

Parameters	Quality Control Specification	Quality control result
Appearance	Clear, colorless	Clear, colorless
Volume	1−2 mL	1 mL
pH	5.0−8.0	7.4
Radiochemistry purity	>95%	>99%
Ethanol	< 5%	< 5%
Sterility	sterile	Pass
Specific activity	NA	12.9‐34.6 GBq/µmol

The in vitro stability of [^6^ ^8^Ga]Ga‐DOTA‐AP9 was assessed in saline, 5% Human Serum Albumin (HSA), and fetal bovine serum (FBS). The radiochemical purity remained above 95% over a 2‐hour incubation period in three kinds of media (Figure 1E and Figure S3), confirming the excellent stability of the tracer under physiological conditions. The lipophilicity of [^6^ ^8^Ga]Ga‐DOTA‐AP9 was determined via the shake‐flask method, yielding a log *p* value of –1.78 ± 0.20, which indicated it was hydrophilic.

### 2.3 In Vitro Biological Evaluation of [^68^Ga]Ga‐DOTA‐AP9

**FIGURE 2:**
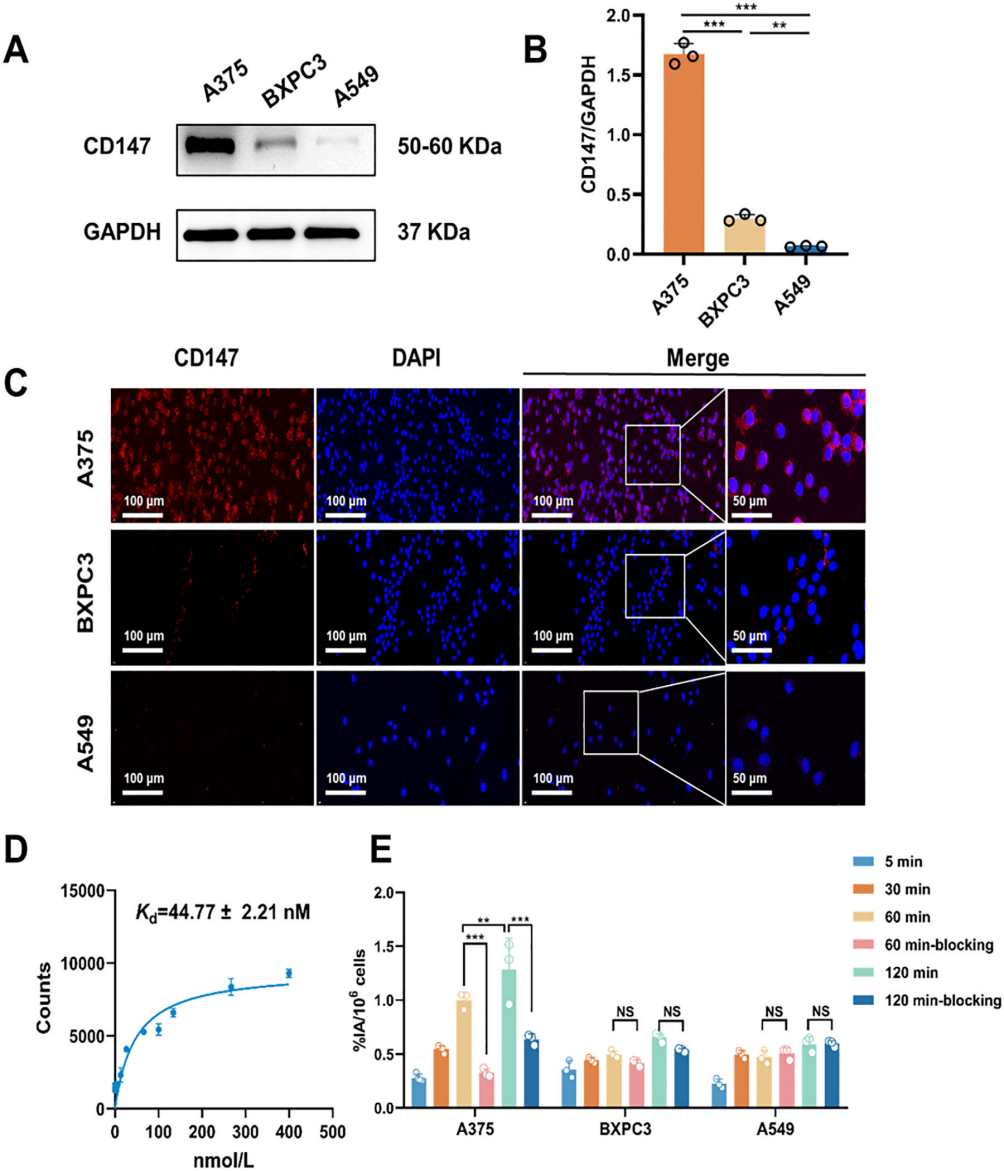
**CD147 expression in different cell lines and in vitro biological evaluation of [^68^Ga]Ga‐DOTA‐AP9**. (A) Western Blot analysis of CD147 protein in tumor cells; (B) Quantitative analysis of CD147 expression normalized to GAPDH (CD147/GAPDH ratios); (C) The IF staining of CD147 on high‐expressing, low‐expressing, and negative‐expressing cells. (D) Binding affinity assay of [^68^Ga]Ga‐DOTA‐AP9 to the CD147 protein. (E) Cell uptake experiments of [^68^Ga]Ga‐DOTA‐AP9 in A375, BXPC3 and A549 cells. Data are presented as the mean ± SD of three independent experiments (n = 3).

Three human tumor cell lines—A375 (malignant melanoma), BXPC3 (pancreatic cancer), and A549 (lung cancer)—were selected for uptake studies. Western blot (WB) and immunohistochemistry (IHC) staining confirmed differential CD147 expression across these lines (Figure 2A, B). Quantitative analysis (normalized to GAPDH) revealed relative CD147 expression levels of 1.67 ± 0.09 in A375, 0.28 ± 0.01 in BXPC3, and 0.05 ± 0.01 in A549 (*P* < 0.01). IHC results were consistent with WB data (Figure 2C), confirming A375 as a CD147‐high, BXPC3 as a CD147‐low, and A549 as a CD147‐negative cell line. These cells were subsequently used for in vitro uptake assays and in vivo xenograft studies.

Saturation binding assays using radio‐ELISA determined the binding affinity of [^6^ ^8^Ga]Ga‐DOTA‐AP9 for CD147, yielding a dissociation constant (*K*_d_) of 44.77 ± 2.21 nM (n = 3; Figure 2D). Cellular uptake studies demonstrated time‐dependent uptake of [^6^ ^8^Ga]Ga‐DOTA‐AP9 in A375 cells, reaching 1.29 ± 0.29%IA/10^6^ cells at 2 h. This uptake was significantly inhibited by co‐incubation with 20 µg of unlabeled DOTA‐AP9, resulting in a ∼50% reduction (0.64 ± 0.05%IA/10^6^ cells, *P* < 0.001). In contrast, BXPC3 and A549 cells showed lower tracer uptake, with maximum values of 0.65 ± 0.04 and 0.59 ± 0.07%IA/10^6^ cells at 2 h, respectively, and no significant blocking effect was observed (Figure 2E).

### 2.4 in vivo Evaluation

**FIGURE 3:**
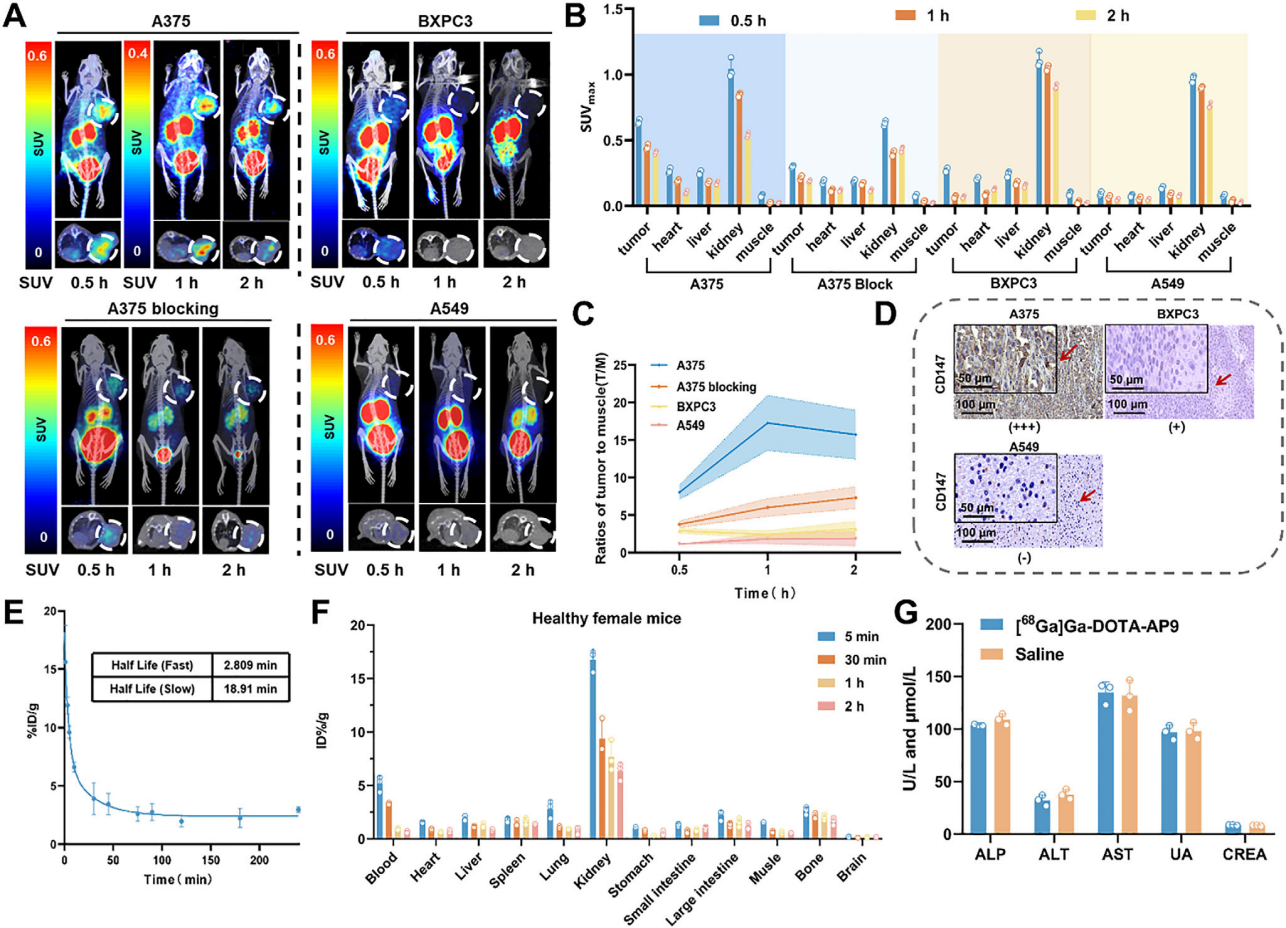
**In vivo evaluation of [^68^Ga]Ga‐DOTA‐AP9**. (A) Micro‐PET/CT images in A375, BXPC3 and A549 xenograft models with/without excess DOTA‐AP9 at 0.5 h, 1 h and 2 h p.i.; (B) Time‐dependent SUV_max_ values in major organs (n = 3); (C) Tumor‐to‐muscle rations at 0.5 h, 1 h and 2 h p.i. (n = 3); (D) Immunohistochemical staining of CD147 in A375, BXPC3 and A549 tumors; (E) Blood pharmacokinetic profile of [^68^Ga]Ga‐DOTA‐AP9 (n = 5); (F) Biodistribution in healthy female mice (n = 3); (G) Renal and liver function markers (ALP, ALT, AST, UA and CREA) following injection of excess [^68^Ga]Ga‐DOTA‐AP9 or saline control (n = 5). Data are presented as the mean ± SD of at least three independent experiments.

[^68^Ga]Ga‐DOTA‐AP9 exhibited rapid clearance from the blood. Pharmacokinetic analysis was best fit by a biphasic model, described by the equation Y = 18.19 + 11.0556·e^−0.2468t^+ 4.7404·e^−0.0367t^, with distribution and elimination half‐lives of 2.81 min and 18.91 min, respectively (Figure 3E). Biodistribution studies in healthy mice revealed predominant renal accumulation, with uptake values of 16.75 ± 1.02%ID/g at 5 min p.i., declining to 6.33 ± 0.81%ID/g at 2 h p.i. (Figure 3F, Table S1). All other organs and tissues showed low tracer retention (<2.8%ID/g at 30 min p.i.).

Micro‐PET/CT imaging in tumor‐bearing mice demonstrated tracer accumulation primarily in the kidneys and urinary system. A375 (CD147‐high) tumors exhibited significantly higher [^6^ ^8^Ga]Ga‐DOTA‐AP9 uptake than BXPC3 (CD147‐low) or A549 (CD147‐negative) tumors, with SUV_max_ values of 0.64 ± 0.02, 0.28 ± 0.02, and 0.09 ± 0.02, respectively, at 30 min p.i. Tumor uptake decreased over time, with SUV_max_ values declining to 0.41 ± 0.02, 0.07 ± 0.01, and 0.05 ± 0.01 at 2 h p.i., respectively. The A375 tumor‐to‐muscle ratio increased over the first hour, reaching a maximum of 17.28 ± 3.69. Co‐injection with an excess of DOTA‐AP9 significantly reduced tracer uptake in both kidneys and A375 tumors (SUV_max_ = 0.30 ± 0.01 at 30 min, 0.22 ± 0.02 at 1 h, and 0.19 ± 0.01 at 2 h p.i.; Figure 3A–C). These imaging findings were consistent with *ex vivo* CD147 IHC staining of the corresponding xenografts (Figure 3D).

### 2.5 Safety and Toxicity Profile

Human radiation dosimetry, extrapolated from mouse biodistribution data using OLINDA/EXM 2.0, identified osteogenic cells as the dose‐limiting organ (0.0179 mGy/MBq), followed by the kidneys (0.0128 mGy/MBq). The estimated effective dose was 1.48 × 10^−^^3^ mSv/MBq (Table S2).

No significant differences in body weight, hematological parameters, or renal and hepatic function markers were observed between mice injected with a 500‐fold higher dose of [^6^ ^8^Ga]Ga‐DOTA‐AP9 and the saline control group (Figure 3G, Figure S4, Table S3). Histopathological examination of major organs via H&E staining revealed no evidence of inflammatory infiltration, necrosis, or other morphological abnormalities. These results supported the favorable safety profile of [^6^ ^8^Ga]Ga‐DOTA‐AP9 and its potential for clinical translation.

### 2.6 Participants, Biodistribution and Safety Evaluation of [^68^Ga]Ga‐DOTA‐AP9

**FIGURE 4:**
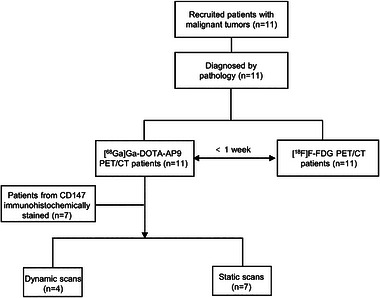
Flow diagram of the clinical study design.

**FIGURE 5:**
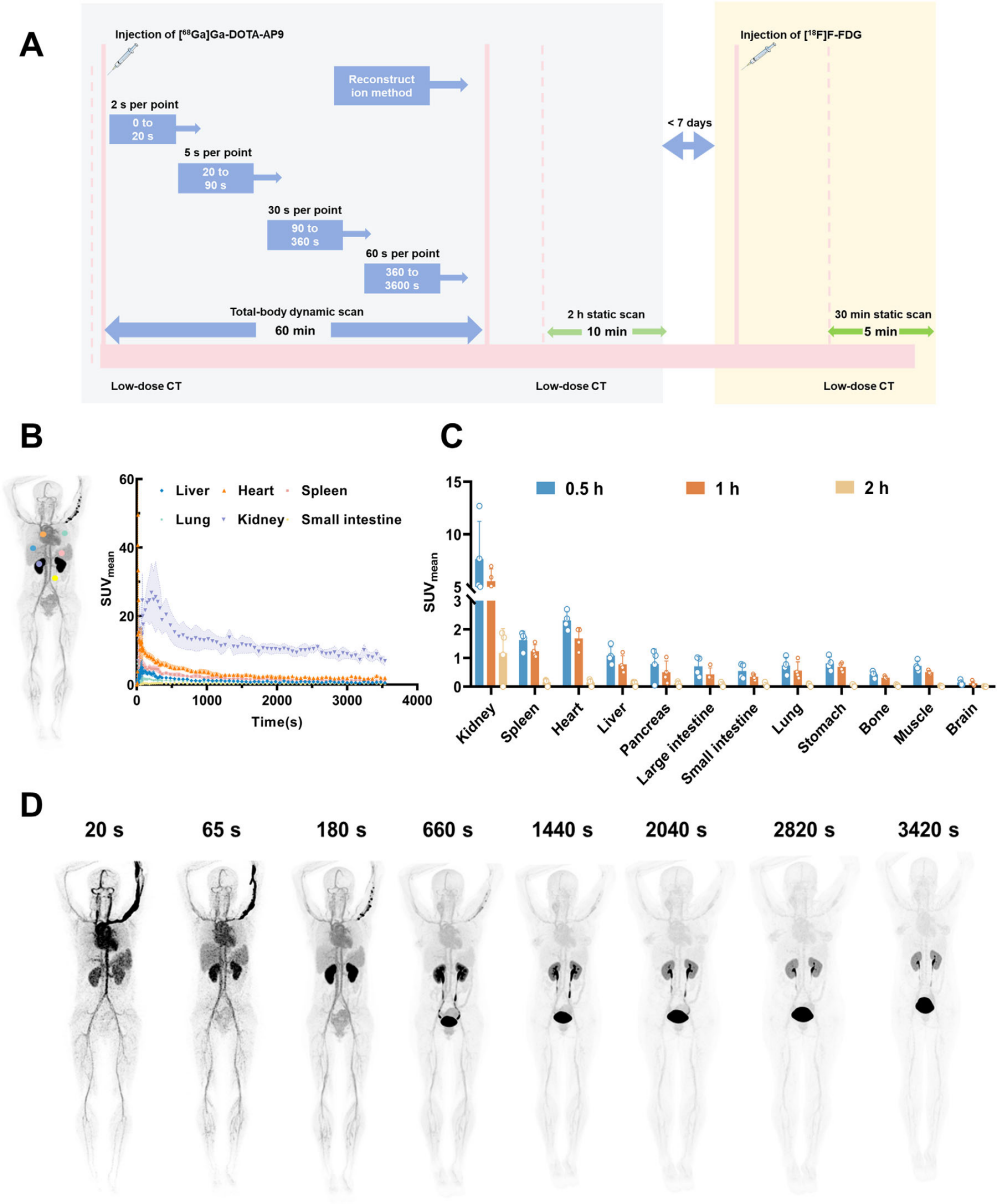
**Clinical evaluation of [^68^Ga]Ga‐DOTA‐AP9**. (A) Dynamic imaging timeline schematic; (B) Tissue biodistribution kinetics of [^68^Ga]Ga‐DOTA‐AP9; (C) Patient biodistribution data from dynamic imaging (n = 4, Patient 01, 04, 07, and 09); (D) Multi‐time‐point dynamic maximum intensity projection (MIP) images from a representative patient.

Eleven participants with three distinct cancer types were enrolled in this translational clinical study using a TB‐PET/CT scanner. Detailed demographic and clinical characteristics are summarized in Table 2. The PET acquisition and dynamic reconstruction protocols are illustrated in Figures 4 and 5A. Four participants underwent dynamic scanning during the first hour p.i., followed by static scans at 2 h p.i. to assess tracer kinetics. A representative dynamic reconstruction is shown in Figure 5D and Video S1.

**TABLE 2:** Basic information of participants.

No	Gender	Age (y)	Dose(MBq)	Clinical Diagnosis	CD147 IHC score	Treatment history	Time interval between treatment and imaging	Clinical staging	Purpose of imaging
01	F	34	199.02	Surgery for melanoma of the right mandible	/	Received dual‐targeted therapy with trametinib plus dabrafenib for 8 months after resection of the primary lesion	25 months	T3bN2M0	Routine follow‐up
02	M	52	81.25	Small cell lung cancer in the right lung	/	No treatment before imaging	/	T4N2M0	Tumor staging
03	F	51	122.84	Adenocarcinoma of the right lung	—	No treatment before imaging	/	T1N2M0	Tumor staging
04	M	66	166.24	Adenocarcinoma of the upper lobe of the left lung	—	No treatment before imaging	/	T4N3M1	Tumor staging
05	M	74	163.32	Anorectal melanoma complicated by liver metastasis	+++	Received 4 cycles of albumin‐bound paclitaxel plus bevacizumab for liver metastasis after resection of the primary anal canal lesion	5 months	TxN3M1	Response evaluation
06	F	72	119.51	Pancreatic cancer with liver metastasis	+	No treatment before imaging	/	T2NxM1	Tumor staging
07	M	61	138.82	Recurrence of malignant melanoma in the right plantar extremity	+++	Received 4 cycles of adjuvant temozolomide after resection of the primary lesion in the right foot	71 months	T4N0M0	Tumor staging
08	M	70	118.25	Malignant melanoma of the right heel extremity	+	No treatment before imaging	/	TxN0M0	Tumor staging
09	F	61	97.01	Small cell lung cancer in the lower lobe of the right lung	—	No treatment before imaging	/	T2N2M1	Tumor staging
10	M	55	52.577	Hepatocellular Carcinoma of the Right Hepatic Lobe	/	No treatment before imaging	/	T2NxM0	Tumor staging
11	M	54	53.28	Hepatocellular Carcinoma of the Right Hepatic Lobe	/	Received 10 cycles of nafinib plus cadonilimab, with hepatic artery chemoembolization during treatment	1 month	T2N0M0	Response evaluation

All participants tolerated the [^6^ ^8^Ga]Ga‐DOTA‐AP9 well, and no adverse events were reported during injection or throughout the 24‐hour follow‐up period. Tissue time–activity curves derived from TB‐PET/CT are shown in Figure 5B. The kidneys showed the highest tracer uptake, with activity decreasing from 30 min to 2 h p.i., consistent with predominant renal clearance. Uptake in all other tissues remained low (SUV_max_ < 2) and cleared rapidly. By 2 h p.i., non‐renal organs exhibited minimal radioactivity, indicating near‐complete tracer clearance from the body. TB‐PET/CT imaging further revealed that absolute tumor uptake peaked at 30 min p.i., whereas the tumor‐to‐muscle ratio reached its maximum at 1 h p.i. (Figure 5C). These biodistribution and kinetic data provide a critical foundation for radiation dosimetry estimation and support the clinical translatability of [^6^ ^8^Ga]Ga‐DOTA‐AP9.

Human radiation dosimetry was estimated using OLINDA/EXM 2.0. The effective dose was 4.20 × 10^−3^ ± 7.90 × 10^−4^ mSv/MBq (Table 3), which is higher than the value extrapolated from mice (1.48 × 10^−3^ mSv/MBq, Table S2) but within the same order of magnitude. The adrenal glands received the highest organ dose (0.0950 mGy/MBq), followed by the kidneys (0.0342 mGy/MBq) and thyroid (0.0339 mGy/MBq).

**TABLE 3:** Human Organ Radiation Dosimetry Estimates for [^68^Ga]Ga‐ DOTA‐AP9.

Organ/tissue	Mean(mGy/MBq)	SD (mGy/MBq)
Adrenals	9.50E‐02	3.69E‐02
Brain	3.88E‐04	1.91E‐04
Breasts	2.50E‐04	5.05E‐05
Esophagus	1.11E‐03	1.79E‐04
Eyes	4.55E‐05	1.24E‐05
Gallbladder Wall	1.31E‐02	9.58E‐03
Left colon	8.55E‐04	1.66E‐04
Small Intestine	1.05E‐03	1.52E‐04
Stomach Wall	2.42E‐03	5.77E‐05
Right colon	3.90E‐04	6.71E‐05
Rectum	2.13E‐03	4.65E‐04
Heart Wall	1.43E‐02	3.09E‐03
Kidneys	3.42E‐02	1.97E‐02
Liver	3.14E‐03	1.08E‐03
Lungs	2.80E‐03	1.35E‐03
Ovaries	1.45E‐04	2.05E‐05
Pancreas	8.63E‐03	5.76E‐03
Salivary Glands	7.21E‐05	4.32E‐06
Red Marrow	3.47E‐04	7.38E‐05
Osteogenic Cells	3.62E‐04	1.48E‐04
Spleen	3.33E‐02	9.07E‐03
Thymus	9.46E‐04	2.02E‐04
Thyroid	3.39E‐02	3.20E‐02
Urinary Bladder Wall	8.93E‐05	1.66E‐06
Uterus	1.67E‐04	4.04E‐06
Total Body	8.23E‐04	1.79E‐04
Effective Dose(mSv/MBq)	4.20E‐03	7.90E‐04

### 2.7 [^68^Ga]Ga‐DOTA‐AP9 PET/CT Imaging in Participants

CD147 is known to be overexpressed in multiple malignancies. Our previous tissue microarray and preclinical studies confirmed high expression in malignant melanoma and pancreatic cancer [21]. Accordingly, this clinical study enrolled participants with pathologically confirmed malignant melanoma, pancreatic cancer, and lung cancer. IHC analysis revealed CD147 positivity in four participants: two with strong staining (+++) and two with weak staining (+). Three participants were CD147‐negative (–). Pathological slides were unavailable for four participants referred from external institutions.

**FIGURE 6:**
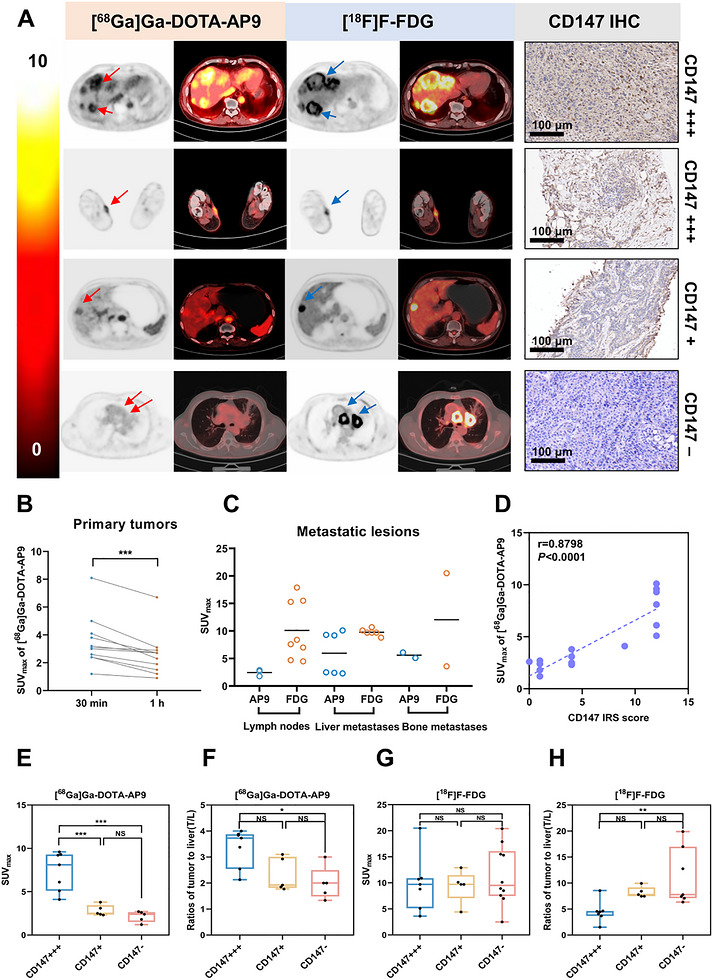
**Comparative [^68^Ga]Ga‐DOTA‐AP9 and [^18^F]F‐FDG PET/CT imaging in participants with varying CD147 expression levels**. (A) Representative images of patient 05 (recurrent melanoma with liver metastases), patient 07 (recurrent melanoma), patient 06 (pancreatic cancer), and patient 04 (lung cancer) at 30 min p.i.; (B) SUV_max_ analysis of [^68^Ga]Ga‐DOTA‐AP9 in primary tumors l at 30 min and 1 h p.i.; (C) SUV_max_ analysis of [^68^Ga]Ga‐DOTA‐AP9 in the metastatic lesions at 30 min and 1 h p.i.; (D) Correlation between CD147 immunoreactive score (IRS) and SUV_max_ values; (D, E) SUV_max_ and tumor‐to‐liver ratio analysis of [^68^Ga]Ga‐DOTA‐AP9 in participants with varying CD147 expression levels; (G, H) SUV_max_ and tumor‐to‐liver ratio analysis of [^18^F]F‐FDG in participants with varying CD147 expression levels.

As shown in Figure 6A, among the two participants with strong CD147 expression (+++), one with anorectal melanoma and liver metastases showed the highest [^6^ ^8^Ga]Ga‐DOTA‐AP9 uptake, with a peak SUV_max_ of 10.1 in the liver metastases at 30 min p.i., compared to an SUV_max_ of 10.7 for [^1^ ^8^F]F‐FDG. The other, with recurrent malignant melanoma in the right plantar region, had a peak [^6^ ^8^Ga]Ga‐DOTA‐AP9 SUV_max_ of 4.1 versus 5.1 for [^1^ ^8^F]F‐FDG. A CD147‐positive pancreatic cancer patient (+) with liver metastasis showed an SUV_max_ of 3.8 for [^6^ ^8^Ga]Ga‐DOTA‐AP9, compared to 12.9 for [^1^ ^8^F]F‐FDG. In contrast, a CD147‐negative lung adenocarcinoma patient had a primary tumor SUV_max_ of only 2.6 with [^6^ ^8^Ga]Ga‐DOTA‐AP9, versus 20.4 with [^1^ ^8^F]F‐FDG.

Notably, [^6^ ^8^Ga]Ga‐DOTA‐AP9 uptake in tumor lesions decreased significantly between 30 min and 1 h p.i. (*P* < 0.001; Figure 6B). Meanwhile, [^68^Ga]Ga‐DOTA‐AP9 exhibited prominent uptake in multiple metastatic lesions, including those in the lymph nodes, liver, and bone (Figure 6C). Furthermore, tracer uptake strongly correlated with the CD147 IRS score (r = 0.8798, *P* < 0.001; Figure 6D). As shown in Figure 6E and F, the mean SUV_max_ values at 30 min p.i. in participants with CD147 (+++), CD147 (+), and CD147 (–) tumors were 7.36 ± 2.24, 2.82 ± 0.63, and 2.14 ± 0.63, respectively. Corresponding tumor‐to‐liver ratios were 3.35 ± 0.73, 2.31 ± 0.64, and 1.99 ± 0.63, respectively. Significant differences in both SUV_max_ and tumor‐to‐liver ratios were observed between the CD147 (+++) and CD147 (–) groups. In contrast, no correlation was found between [^1^ ^8^F]F‐FDG uptake and CD147 expression, either in terms of SUV_max_ or tumor‐to‐liver ratios (Figure 6G, H).

### 2.8 Histopathological and Correlation Analysis

**FIGURE 7:**
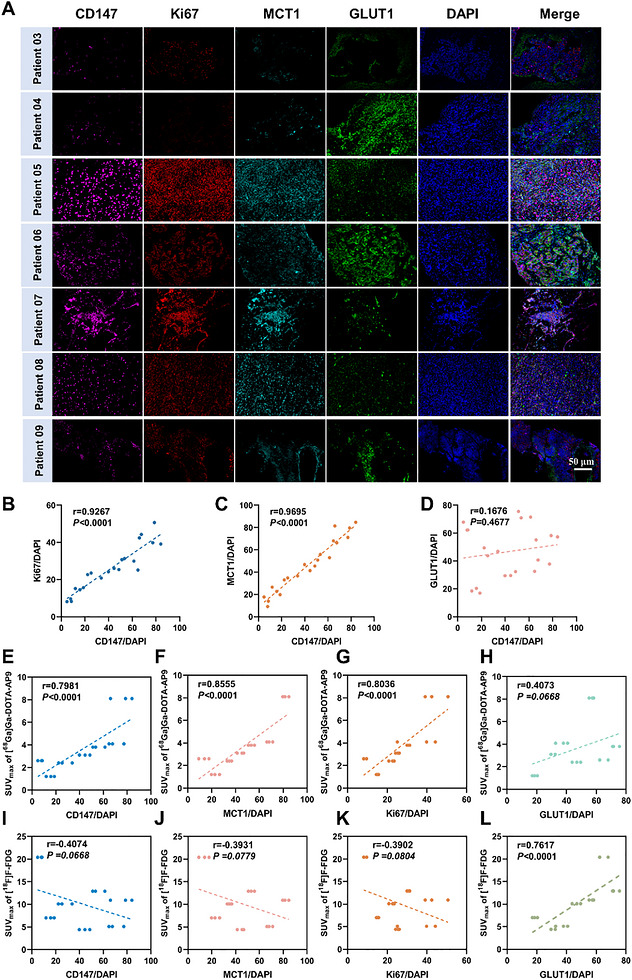
**Analysis of the MxIF staining results for tumor tissue sections from participants numbered 03–09**. (A) MxIF images of tumor tissues of participants. Staining was performed for CD147 (pink), Ki67 (red), MCT1 (cyan), GLUT1 (green), and DAPI (blue). Original magnification: ×20 (scale bar = 50 µm); (B–D) Correlation analysis between CD147 and Ki67, MCT1, and GLUT1; (E–H) Correlation analysis between the SUV_max_ of [^68^Ga]Ga‐DOTA‐AP9 at 30 min p.i. and CD147/MCT1/Ki67/GLUT1 expression; (I–L) Correlation analysis between the SUV_max_ of [^18^F]F‐FDG at 30 min p.i. and the CD147/MCT1/Ki67/GLUT1 expression. Data are presented as the mean ± SD of three independent experiments (n = 3).

As shown in Figure 7A, CD147 expression was high (+++) in tumor sections from participants 05 and 07, moderate (+) in participants 06 and 08, and low (–) in participants 03, 04, and 09. Expression of Ki67 and MCT1 closely mirrored that of CD147. In contrast, GLUT1 exhibited an independent expression profile, with particularly high levels in participants 04 and 06.

Correlation analysis revealed strong positive associations between CD147 expression and Ki67 (r = 0.9267, *P* < 0.001; Figure 7B) and MCT1 (r = 0.9695, *p* < 0.001; Figure 7C). No significant correlation was observed between CD147 and GLUT1 (r = 0.1676, *P* = 0.4677; Figure 7D). The tumor SUV_max_ of [^6^ ^8^Ga]Ga‐DOTA‐AP9 correlated positively with CD147 (r = 0.7981, *P* < 0.001), MCT1 (r = 0.8555, *p* < 0.001), and Ki67 (r = 0.8036, *P* < 0.001), but not with GLUT1 (*P* = 0.0668; Figure 7E–H). Conversely, [^1^ ^8^F]F‐FDG SUV_max_ correlated positively with GLUT1 (r = 0.7617, *P* < 0.001; Figure 7L), but not with CD147 (*p* = 0.0668), MCT1 (*P* = 0.0779), or Ki67 (*P* = 0.0804; Figure 7I–K).

## 3 Discussion

CD147, a transmembrane glycoprotein overexpressed in numerous malignancies, has emerged as a promising target for cancer theranostics due to its multifaceted role in promoting tumor progression, including facilitation of extracellular matrix remodeling, angiogenesis through induction of MMPs and VEGF [6, 28], and metabolic reprogramming via its molecular chaperone activity for monocarboxylate transporters (MCTs) [29, 30]. While the clinical relevance of CD147 is underscored by emerging targeted therapies, such as the radioimmunotherapeutic agent [^131^I]I‐Metuximab (Licartin) and CD147‐directed CAR‐T/NK cell therapies [14, 15, 16, 17, 18], this field has been notably hampered by the absence of a noninvasive method to quantitatively assess CD147 expression in vivo. This critical gap has significantly hindered patient stratification for CD147‐targeted therapies and the ability to reliably monitor pharmacodynamic treatment responses. Herein, we report the first‐in‐human application of [^68^Ga]Ga‐DOTA‐AP9, the first CD147‐targeted peptide‐based PET probe, which establishes a pioneering platform for the noninvasive visualization and quantification of CD147 expression in human cancers.

The development of [^68^Ga]Ga‐DOTA‐AP9 addresses a critical unmet need in molecular theranostics. While its measured affinity (*K*_d_ = 44.77 ± 2.21 nM) is moderate compared with that of the previously reported radioiodine‐labeled antibody ([^125^I]I‐anti‐CD147: *K*_d_ = 6.34 nM) [7] and nanobody ([^124^I]I‐NB147: *K*_d_ = 10.30 nM) [21], it represents a notable affinity achievement for a peptide‐based probe. More importantly, [^68^Ga]Ga‐DOTA‐AP9 exhibited an optimal pharmacokinetic profile distinct from antibodies, characterized by rapid blood clearance and predominantly renal excretion. This profile enabled high tumor‐to‐background ratios within a short timeframe, a significant advantage for clinical workflow. The specificity of [^68^Ga]Ga‐DOTA‐AP9 was rigorously validated through a comprehensive series of in vitro and in vivo studies, demonstrating high and blockable CD147‐dependent uptake in CD147‐positive models and minimal in CD147‐negative controls.

The comprehensive evaluation of cellular and xenograft tumor models demonstrated the specificity of [^68^Ga]Ga‐DOTA‐AP9 for CD147, with high and blockable uptake in CD147‐positive A375 cells and xenograft tumors, and low uptake in CD147‐negative A549 cells/tumors. The specific uptake in A375 was significantly higher than that in BXPC3 and A549 cells. As a linear small peptide, [^68^Ga]Ga‐DOTA‐AP9 showed favorable pharmacokinetics, with rapid clearance from blood and non‐target tissues via renal excretion, producing high tumor‐to‐background contrast in CD147‐positive xenografts and low radiation dosimetry (effective dose: 1.48× 10^−3^ mSv/MBq). The safety in mice was verified by the administration of an excess dose of [^68^Ga]Ga‐DOTA‐AP9. Together, these features make [^68^Ga]Ga‐DOTA‐AP9 an optimal molecular imaging agent for clinical translation.

The translation of [^68^Ga]Ga‐DOTA‐AP9 to a first‐in‐human trial marks a seminal advance. Clinical translation is supported by excellent safety profiles, with no observed toxicity and lower radiation dosimetry than [^1^ ^8^F]F‐FDG and [^131^I]I‐Meplazumab [20]. Although human dosimetry revealed a higher effective dose (4.20 × 10^−3^ mSv/MBq) than preclinically estimated (1.48× 10^−3^ mSv/MBq). The likely explanation is that the human patients involved in radiation dose calculation underwent dynamic scanning without bladder voiding, while the murine biodistribution study did not collect uptake values for the bladder or urine. Nevertheless, the radiation dose estimated from mouse data was still deemed applicable for preclinical imaging safety evaluation. Furthermore, the low overall radiation dose delivered by the uniform use of total‐body (TB) PET/CT (194 cm‐axial field of view) across all enrolled patients contributed to the high safety of this clinical trial.

Consistent with preclinical data, [^6^ ^8^Ga]Ga‐DOTA‐AP9 demonstrated rapid metabolism in subjects. Dynamic imaging revealed that uptake in most organs (except the kidneys) peaked within the first 2 min p.i., followed by gradual clearance and maintenance at low levels. It showed the highest uptake in the kidneys, which increased briefly before progressively decreasing. Compared with [^131^I]I‐Meplazumab, for which human biodistribution data in healthy volunteers were reported, [^6^ ^8^Ga]Ga‐DOTA‐AP9 showed low uptake in non‐target organs excepting for the kidneys, and fast clearance [20]. Dynamic scans indicated that good contrast could be observed as early as 1440 s p.i. [^6^ ^8^Ga]Ga‐DOTA‐AP9 demonstrated CD147‐specific uptake across cancer types, with peak accumulation at 30 min p.i. Considering tumor uptake and non‐target organ metabolism, 30 min p.i. was selected as the optimal static scanning time point. Representative T‐B PET/CT MIP images of three subjects, shown in Figure S5, confirmed the high contrast.

As a vital organ, the liver's probe uptake serves as a reference standard in numerous studies for lesion identification. In this study, the ROIs in the liver were drawn, and the uptake was quantified. The tumor‐to‐liver ratios were calculated. Tumor sections from seven participants were collected, and CD147 expression was assessed by IHC staining. Most importantly, [^68^Ga]Ga‐DOTA‐AP9 uptake in tumors strongly correlated with CD147 expression levels across models and participants, as well as with tumor‐to‐liver ratios. Suggesting its utility for noninvasive CD147 quantification.

[^18^F]F‐FDG is the most used PET tracer in clinical practice, which enters cells through GLUTs, especially for GLUT1. In this study, [^18^F]F‐FDG was used as the standard. The distinct imaging patterns observed between [^6^ ^8^Ga]Ga‐DOTA‐AP9 and [^1^ ^8^F]F‐FDG further underscore their complementary value. In one case, [^1^ ^8^F]F‐FDG showed peripheral uptake with central photopenia—a pattern likely consistent with hypoxia‐mediated suppression—while [^6^ ^8^Ga]Ga‐DOTA‐AP9 accumulated throughout the tumor core. This observation, coupled with evidence that CD147 promotes hypoxic survival via HIF‐1α and MCT1, suggests that CD147‐targeted PET may provide unique biological information unaffected by tumor hypoxia [31].

Ki67 is a proliferation marker; MCT1 primarily mediates the efflux of lactate produced by glycolysis in tumor cells, thereby promoting tumor progression; GLUT1 is the most critical protein responsible for the increased glucose metabolic rate in malignant tumor cells and is the most extensively studied glucose transporter, and the most widely used PET tracer ([^18^F]F‐FDG) in clinical practice reflects the glucose metabolism mediated by GLUTs. Su et al. demonstrated that CD147 and GLUT1 were colocalized in the cytoplasm of human melanoma A375 cells, that silencing CD147 downregulated GLUT1 expression, suppressed tumor growth, and reduced [^18^F]F‐FDG uptake [32]. CD147 also contributes to tumor glycolysis by co‐expressing and interacting with MCT1 and MCT4, thereby regulating lactate transport, promoting tumor progression and metastasis, facilitating immune evasion, and modulating PD‐1 blockade efficacy [33, 34, 35]. Given the multifaceted roles of CD147 in tumors, multiplex immunofluorescence analysis of clinical specimens was performed to detect Ki67, MCT1, and GLUT1 expression, further exploring the potent value of [^68^Ga]Ga‐DOTA‐AP9 in the clinic. CD147 expression revealed strong correlations with both Ki67 (proliferation marker) and MCT1, but not with GLUT1. This contrasts with reported preclinical studies, which may highlight potential differences between model systems and clinical tumors [32]. The SUV_max_ of [^68^Ga]Ga‐DOTA‐AP9 was correlated with the expression levels of Ki67 and MCT1, while the SUV_max_ of [^1^ ^8^F]F‐FDG did not demonstrate such correlations. These results indicated that CD147‐targeted imaging may be an appropriate complement to [^1^ ^8^F]F‐FDG in the precision theranostics of cancers.

Our preliminary experience in melanoma participants supports the clinical potential of [^6^ ^8^Ga]Ga‐DOTA‐AP9, particularly given CD147's established roles in metastasis, glycolysis, and immune evasion in this disease^4^ ^1^. Larger studies are warranted to fully define its utility in diagnosis, recurrence monitoring, and therapy response assessment. While [^6^ ^8^Ga]Ga‐DOTA‐AP9 demonstrates high CD147 affinity and reliable target expression correlation, its rapid metabolism and suboptimal tumor retention currently limit therapeutic applications. Future efforts will focus on structural optimization to enhance pharmacokinetic properties, potentially enabling a CD147‐targeted theranostic platform.

In conclusion, we have developed and clinically translated [^68^Ga]Ga‐DOTA‐AP9, the first‐in‐class CD147‐targeted PET probe. Its high specificity, favorable safety profile, and proven ability to noninvasively quantify CD147 expression in vivo position it as a transformative tool for patient selection, therapy monitoring, and enhancing our understanding of CD147 biology in human cancers. Its complementary information to [^18^F]F‐FDG suggests its immediate utility in precision oncology, particularly in cancers like melanoma, where it plays a pivotal role in metastasis and immune evasion. This study not only provides a novel diagnostic agent but also paves the way for a new generation of CD147‐directed theranostics.

## 4 Conclusions

In this study, we developed a CD147‐targeted peptide probe, [^68^Ga]Ga‐DOTA‐AP9, and subsequently performed the first‐in‐human study. Preclinical validation confirmed its binding affinity and specificity for CD147. The initial clinical trial demonstrated high uptake in CD147‐positive tumors, with strong positive correlations between tumor uptake and CD147, Ki67, and MCT1 expression levels. [^68^Ga]Ga‐DOTA‐AP9 shows promise as a complementary diagnostic tool to [^18^F]F‐FDG for CD147‐positive malignancies and may help guide CD147‐targeted therapeutic decision‐making.

## 5 Experimental Section

### 5.1 General

Human Serum Albumin (HSA, 20%; CSL Behring, Bern, Switzerland), Dulbecco's modified Eagle's medium (DMEM, Invitrogen, USA), Roswell Park Memorial Institute‐1640 (RPMI‐1640, HyClone, USA), and other reagents were commercially purchased. Mice were purchased from Beijing Vital River Laboratory Animal Technology Co., Ltd. (Beijing, China). A [^68^Ge]Ge/[^68^Ga]Ga generator was purchased from Isotope Technologies (Germany). Sep‐Pak C18 Light Cartridge was purchased from Waters (Cat No. WAT023501, Germany). The radiochemical purity was determined by radioactive high performance liquid chromatography (radio‐HPLC; HPLC 1200, Agilent Technologies, USA) using a gradient elution of water (containing 0.1% trifluoroacetic acid; 95%–65% from 0–10 min; 65%–5% from 10–20 min) and acetonitrile (containing 0.1% trifluoroacetic acid; 5%–35% from 0–10 min; 35%–95% from 10–20 min). Micro‐PET/CT imaging was performed using a Super Nova PET/CT system (PINGSENG Healthcare Inc., Shanghai, China). Additionally, a total‐body PET/CT scanner (uEXPLORER, United Imaging Healthcare, Shanghai, China) with a 194‐cm axial field of view was utilized.

### 5.2 Cells and Tumor Models

During the reported studies, A375 and BXPC3 cells were selected as CD147‐positive models with varying expression levels, while A549 cells served as CD147‐negative cells [19, 36, 37]. All cell lines were obtained from Procell Life Science & Technology Co., Ltd. (Wuhan, China). Cells were cultured at 37 °C in a humidified incubator with 5% CO_2_, in medium supplemented with 15% fetal bovine serum (FBS) and 1% penicillin‐streptomycin. A375 cells were incubated in DMEM medium, while BXPC3 and A549 cells were cultured in RPMI‐1640 medium.

Female BALB/c nude mice (4‐5 weeks old, 18–20 g) were housed under specific pathogen–free (SPF) conditions. For xenograft tumor establishment, 6 × 10^6^ tumor cells suspended in 150 µL of sterile phosphate‐buffered saline (PBS) were subcutaneously injected into the right flank of each mouse. Imaging was performed when tumor volumes reached approximately 6–8 mm in diameter. All animal procedures were conducted in compliance with the guidelines of the Institutional Animal Care and Use Committee (IACUC) and were approved by the Ethics Committee of Peking University Cancer Hospital and Institute (EAEC 2023‐18).

### 5.3 Synthesis of Precursor DOTA‐AP9

The precursor DOTA‐AP9 was synthesized via automated solid‐phase peptide synthesis by China Peptides company (Shanghai, China), with detailed procedures provided in the Supporting Methods. Following synthesis, DOTA‐AP9 was purified by high‐performance liquid chromatography (HPLC) and characterized by electrospray ionization mass spectrometry (ESI‐MS).

### 5.4 Molecular Docking of AP9 and DOTA‐AP9 with CD147

The predicted structures of CD147 were predicted using AlphaFold 3.0 [38]. The protonation states of AP9 and DOTA‐AP9 were adjusted to pH 7.4, and their 3D structures were generated using Open Babel [39]. Receptor and ligand preparation, including parameter assignment, was performed with AutoDock Tools. Docking grids were constructed using AutoGrid for site mapping, and molecular docking simulations were carried out with AutoDock Vina (v1.2.0) [40]. The top‐ranked docking pose was selected for interaction analysis. Protein‐ligand interaction diagrams were rendered in PyMOL. In these visualizations, the CD147 protein was depicted as a slate cartoon, ligands as cyan sticks, and binding‐site residues as magenta sticks. Nonpolar hydrogen atoms were omitted for clarity. Hydrogen bonds, ionic interactions, and hydrophobic contacts were represented by yellow, magenta, and green dashed lines, respectively.

### 5.5 Radiolabeling and Quality Control

[^68^Ga]GaCl_3_ was obtained by eluting a [^68^Ge]Ge/[^68^Ga]Ga generator with 4 mL of 0.05 M hydrochloric acid. The last 3 mL of eluate was mixed with 180 µL of 1 M sodium acetate and 40 µg of DOTA‐AP9 (20 µL, 2 mg/mL), followed by incubation at 90 °C for 15 min. After cooling to room temperature, the radiolabeling yield was determined, and the reaction mixture was purified using a Sep‐Pak C18 Light Cartridge (preconditioned with 5 mL of ethanol and 5 mL of water). The final product, [^6^ ^8^Ga]Ga‐DOTA‐AP9, was eluted with 0.5 mL of 80% ethanol, sterilized by filtration, and diluted with saline. Quality control tests were performed to determine radiochemical purity (RCP), specific activity, pH, sterility, and apyrogenicity.

### 5.6 In Vitro Stability and Partition Coefficient Determination

The in vitro stability of [^68^Ga]Ga‐DOTA‐AP9 was assessed by incubating the radiotracer (14.8 MBq) in 200 µL of saline, 5% HSA or fetal bovine serum (FBS) at 37°C. Samples were analyzed by radio‐HPLC at 0, 0.5, 1, and 2 h post‐incubation to determine the RCP.

Lipophilicity, expressed as the logarithm of the n‐octanol/PBS partition coefficient (log P), was measured using a shake‐flask method. Briefly, equal volumes of 0.01 M PBS (pH 7.0) and n‐octanol were pre‐saturated and allowed to separate overnight. Subsequently, 590 µL of the PBS phase was mixed with 10 µL of [^6^ ^8^Ga]Ga‐DOTA‐AP9, followed by the addition of 600 µL of the n‐octanol phase. The mixture was vortexed vigorously and centrifuged at 3,000 rpm for 5 min. Aliquots (50 µL; n  =  5) were then collected from each phase, and the radioactivity was quantified using an automated γ‐counter. The log *p* value was calculated from the ratio of counts in the n‐octanol phase to those in the PBS phase. The study was repeated three times.

### 5.7 Cell Uptake and Binding Affinity Assay

For cell uptake studies, A375, BXPC3, and A549 cells were plated in 24‐well plates at a density of 2 × 10^6^ cells per well in 500 µL of medium and allowed to adhere for 24 h. The medium was then replaced with serum‐free medium. Cells were incubated with [^68^Ga]Ga‐DOTA‐AP9 (74 kBq) at 37°C. To assess the non‐specific binding, 10 µg of DOTA‐AP9 was added. At designated time points (5, 30, 60, and 120 min), the medium was removed, and the cells were washed twice with cold PBS and subsequently lysed with cold 1 M NaOH. The associated radioactivity was measured using a γ‐counter, and the results were expressed as the percentage of total added dose per 10^6^ cells (%ID/10^6^ cells).

The binding affinity of [^6^ ^8^Ga]Ga‐DOTA‐AP9 for CD147 was determined using a radioligand binding assay. A 96‐well plate was coated with 100 µL of CD147 protein (1 µg/mL; Cat.10186‐H02H, Sino Biological, Beijing, China) in ELISA Coating Buffer (Cat. C1055; Solarbio, Beijing, China) and incubated overnight at 4 °C. The plate was then washed five times with ice‐cold PBST (0.01 M PBS, pH 7.4, containing 0.2% Tween‐20) and blocked with 200 µL of 5% skim milk for 2 h at 37°C. Serial dilutions of [^6^ ^8^Ga]Ga‐DOTA‐AP9 in saline (12 concentrations ranging from 0.00037 to 11.1 MBq/mL) were prepared, and 50 µL of each concentration was added to the wells and incubated for 2 h at 37°C. After incubation, the wells were washed, excised, and the bound radioactivity was quantified using an automated γ‐counter. The binding data were analyzed by nonlinear regression using GraphPad Prism 8.0 software to determine the equilibrium dissociation constant (*K*_d_).

### 5.8 Pharmacokinetics and Biodistribution

The pharmacokinetics profile of [^68^Ga]Ga‐DOTA‐AP9 was evaluated in healthy female BALB/c mice (4 weeks old, 18–20 g; n = 5). Each mouse was intravenously administered with the radiotracer (0.74 MBq in 200 µL). Blood samples were collected from the orbital venous plexus at 1, 3, 5, 10, 20, 30, 45, 60, 75, 90, 120, 180, and 240 minutes post‐injection (p.i.). Each sample was accurately weighed, and the radioactivity was measured using an automated γ‐counter. The blood pharmacokinetic parameters of [^6^ ^8^Ga]Ga‐DOTA‐AP9 were analyzed by applying a biphasic decay model. All data were decay‐corrected and expressed as the percentage of injected dose per gram of tissue (%ID/g).

For the biodistribution study, twelve healthy female BALB/c mice were randomly divided into 4 groups (n = 3 per group) and intravenously injected with [^68^Ga]Ga‐DOTA‐AP9 (200 µL, 0.74 MBq). The mice were euthanized at 5 min, 30 min, 1 h, and 2 h p.i. Major organs and tissues were then collected, weighed, and measured for radioactivity. The results were expressed as the %ID/g. Based on the biodistribution data obtained from mice, the human radiation dosimetry of [^68^Ga]Ga‐DOTA‐AP9 was estimated using OLINDA/EXM 2.0 software (Hermes).

### 5.9 Micro‐PET/CT Imaging

A375, BXPC3, and A549 tumor‐bearing mice (n = 3 per model) were intravenously injected with [^68^Ga]Ga‐DOTA‐AP9 (200 µL, 7.4 MBq). Micro‐PET/CT imaging was performed at 0.5 h, 1 h and 2 h p.i. under anesthesia (1.5% isoflurane in oxygen, 0.5 L/min) to assess binding specificity. For the blocking group, mice were co‐injected with 100 µg of DOTA‑AP9. After image reconstruction, regions of interest (ROIs) were drawn for the tumor, heart, liver, kidneys and muscle. The maximum standardized uptake values (SUV_max_) for each ROI were quantified using Avatar 3.0 software (PINGSENG Healthcare Inc., Shanghai, China).

### 5.10 Radiation Toxicity

A preliminary toxicity study was conducted in ten healthy female BALB/c mice (4 weeks old, 18–20 g), which were allocated into an experimental group (n = 5) and a control group (n = 5). Mice in the experimental group received a single intravenous injection of [^6^ ^8^Ga]Ga‐DOTA‐AP9 at a dose 500‐fold higher than the clinical imaging dose (37 MBq in 200 µL), while the control group received an equal volume of saline. Body weight and blood samples (collected via the orbital venous plexus) were monitored for complete blood count analysis at baseline (day 0) and on days 1, 2, 3, 5, and 7 p.i. On day 7, all mice were euthanized. Blood samples were allowed to clot at room temperature for 25–30 min and then centrifuged at 3,000 rpm for 10–15 min to obtain plasma for liver and kidney function biochemical assays. Furthermore, major organs—including the heart, liver, spleen, lungs, kidneys, stomach, small intestine, large intestine, brain, and skeletal muscle—were harvested from both groups for histopathological examination.

### 5.11 Clinical Trial Approval and Participant Preparation

This translational clinical study was approved by the Ethics Committee of Beijing Cancer Hospital (No. 2024YJZ142) and registered at ClinicalTriails.gov (NCT06720298). The study was conducted following the guidelines of the Declaration of Helsinki, and written informed consent was obtained prior to the study.

Eligible participants were aged 18–75 years with histologically confirmed malignant tumors and were enrolled prior to pharmacological treatment or surgery. Key exclusion criteria included severe hepatic or renal dysfunction, pregnancy, and lactation. Eleven eligible participants were enrolled in the study (demographic and clinical characteristics were summarized in Table 3). A uEXPLORER total‐body PET/CT scanner (United Imaging Healthcare, Shanghai, China) was selected for this pilot study due to its ability to simultaneously cover the entire body and its high detection sensitivity. This technology enables a reduction in the total radiation dose administered and facilitates translational research for novel radiopharmaceuticals.

### 5.12 [^68^Ga]Ga‐DOTA‐AP9 Total‐Body PET/CT Imaging

[^68^Ga]Ga‐DOTA‐AP9 was prepared under Good Laboratory Practice (GLP) conditions using a compliant hot cell (NMC Ga‐68, Tema Sinergie, S.p.A, Italy) and passed all quality control tests prior to administration. Participants received an intravenous injection of 5 mL of [^68^Ga]Ga‐DOTA‐AP9 (119.28 ± 46.53 MBq) in saline. Imaging was performed using a 194‐cm axial field‐of‐view total‐body PET/CT system. A low‐dose CT scan was performed for attenuation correction prior to injection. Subsequently, four participants with oligofocal lesions underwent a total‐body dynamic PET scan immediately after intravenous injection of [^68^Ga]Ga‐DOTA‐AP9 (dose: 1.85–3.7 MBq/kg), which continued for 60 min. All participants additionally underwent a static T‐B PET/CT scan at 120 min p.i., with an acquisition time of 10 min.

Within one week before or after the [^68^Ga]Ga‐DOTA‐AP9 scan, all participants also underwent a standard [^18^F]F‐FDG T‐B PET/CT scan for comparison. The [^18^F]F‐FDG scan was acquired at 40 min p.i., after confirmation that the blood glucose level in all participants was < 160 mg/dL before tracer administration.

### 5.13 PET/CT Imaging Reconstruction

Image reconstruction was performed for three distinct datasets: (1) dynamic images covering 0–60 min, (2) static images reconstructed from 55–60 min, and (3) delayed static images reconstructed from the 120 min scan. The dynamic data were reconstructed into 88 time frames as follows: 2 s per frame (0–20 s), 5 s per frame (20–90 s), 30 s per frame (90–360 s), and 60 s per frame (360–3600 s). All frames were reconstructed using an ordered subset expectation maximization (OSEM) algorithm (4 iterations, 20 subsets) with point‐spread function modeling and time‐of‐flight (TOF) correction.

Time‐activity curves were generated by placing regions of interest (ROIs) on tumors and major organs across all 88 dynamic frames. Static images were reconstructed from list‐mode data using vendor‐provided software (United Imaging) with an iterative algorithm (20 subsets, 4 iterations) incorporating TOF but without point‐spread function correction.

### 5.14 Biodistribution and Dosimetry Evaluation in Humans

All [^68^Ga]Ga activity measurements were decay‐corrected to the time of injection and normalized to the administered activity. Data processing was performed using the vendor's Multi‐Modality Workplace software (United Imaging). To assess biodistribution, ROIs were manually drawn on the largest transverse cross‐section of major organs and tissues visible on the 60‐min static scan. Normal organs and tissues analyzed included the kidneys, aorta, salivary glands, esophagus, colon, liver, pancreas, intestine, thyroid, spleen, adrenal glands, lacrimal glands, gallbladder, lung, skin, brain, bone marrow, etc. The maximum standardized uptake value (SUV_max_) was automatically extracted from each ROI for subsequent analysis and comparison. SUV_max_ was calculated as follows:

$$\mathrm{SU}V_{\max}=r/\left(\frac{a^{\prime }}{w}\right)$$

where *r* denotes the peak radioactivity concentration (kBq/mL) within the ROI, *a′* was the decay‑corrected administrated activity (kBq), and *w* was the patient's body weight (g).

### 5.15 Pathological Studies

Formalin‐fixed, paraffin‐embedded (FFPE) tumor sections were obtained from seven enrolled participants. Tumor sections were subjected to IHC and multiplex immunofluorescence (MxIF) staining. Correlation analyses were conducted between the SUV_max_ of [^6^ ^8^Ga]Ga‑DOTA‑AP9, [^1^ ^8^F]F‐FDG and the expression levels of CD147, proliferation cell nuclear antigen (Ki67), monocarboxylate transporter 1 (MCT1), and glucose transporter 1 (GLUT1). Detailed staining protocols were provided in the Supporting Methods.

All stained samples were evaluated independently by three board‐certified pathologists. CD147 expression was quantified using the immunoreactivity score (IRS) system [41], which combines staining intensity (0  =  negative, 1  =  weak, 2  =  moderate, 3  =  strong) and the percentage of positive cells (0  =  <5%, 1  =  5–25%, 2  =  25–50%, 3  =  50–75%, 4  =  >75%). The final IRS was calculated by multiplying the intensity and percentage scores, with expression levels categorized as negative (0–1), low (2–4), medium (5–8), or high (≥9).

### 5.16 Statistical Analysis

Statistical analyses were performed using SPSS 24.0 and GraphPad Prism 8.0. Parametric continuous variables were expressed as mean ± standard deviation (SD). A mixed‐effects model with subjects as random effects was applied for longitudinal analyses. Independent‐sample t‐tests were used for two‐group comparisons, and one‐way ANOVA or Kruskal–Wallis tests, with appropriate post hoc corrections, were used for multi‐group comparisons. Relationships between PET quantitative values (SUV_max_ of [^6^ ^8^Ga]Ga‑DOTA‑AP9 and [^1^ ^8^F]F‐FDG) and protein expression (CD147, Ki67, MCT1, and GLUT1 by IHC/MxIF staining) were analyzed using logistic regression. The Spearman correlation coefficient was used to assess the association between the SUV_max_ values and IHC scores. A two‐sided *p*‐value< 0.05 was considered statistically significant.

### 5.17 Study Approval

All the animal experiments were approved by the Ethics Committee of Peking University Cancer Hospital (approval number: EAEC 2023‐18). The clinical study was approved by the Ethics Committee of Peking University Cancer Hospital (approval number: 2024YJZ142), and this study was registered on ClinicalTrials.gov (NCT06720298). Written informed consent was obtained from all participants.

## Author Contributions

**T.L**. designed the experiments. **X.M**. performed most of the preclinical experiments and analyzed data. **X.M**. and **R.G**. supervised the clinical studies and analyzed data. **Y.S**., **Z.Z**., and **X.M**. performed the imaging and reconstruction studies. **X.M**. and **H.H**. assisted with animal studies and data analysis. **D.N**. performed the IHC of clinical samples. **X.M**. prepared the figures. **T.L**., **X.M**., **H.Z**., and **Z.Y**. wrote the manuscript with contributions from the other authors. **T.L**., **Z.Y**., **H.Z**., and **R.G**. conceived the project and supervised the research. All authors reviewed and approved the final version of the manuscript.

## Conflicts of Interest

The authors declare no conflicts of interest.

## Supporting information




**Supporting File 1**: advs75795‐sup‐0001‐SuppMat.doc.

**Supporting File 2**: advs75795‐sup‐0002‐VideoS1.mp4.

## Data Availability

The data that support the findings of this study are available from the corresponding author upon reasonable request.
