# Supplementary material for: The Development and Pilot Clinical Study of CD147 Targeted Antagonistic Peptide Probe for Tumor Imaging
Source: Adv Sci (Weinh). 2026 May 26:e22550. Online ahead of print. doi: 10.1002/advs.202522550 (PMC13335849; doi:10.1002/advs.202522550)
Supplement: Supplementary file 1 — Supporting File 1: advs75795‐sup‐0001‐SuppMat.doc. [file ADVS-9999-e22550-s002.doc]

Supplementary Information for

**The development and pilot clinical study of CD147 targeted antagonistic peptide probe for tumor imaging**

Xiaokun Ma^1#^, Rui Guo^1#^, Dongfeng Niu^2#^, Yufei Song^1^, Zhenyao Zhang^1^, Xiangxi Meng^1^, Lili Mao^3^, Haifeng Huang^4^, Hua Zhu^1*^, Zhi Yang^1*^, Teli Liu^1*^

*^1^ State Key Laboratory of Holistic Integrative Management of Gastrointestinal Cancers, Beijing Key Laboratory of Research, Investigation and Evaluation of Radiopharmaceuticals, NMPA Key Laboratory for Research and Evaluation of Radiopharmaceuticals (National Medical Products Administration), Department of Nuclear Medicine, Peking University Cancer Hospital & Institute, Beijing 100142, China*

*^2^ Key laboratory of Carcinogenesis and Translational Research (Ministry of Education), Beijing Key Laboratory of Research, Department of Pathology, Peking University Cancer Hospital & Institute, Beijing 100142, China*

*^3^ Key Laboratory of Carcinogenesis and Translational Research (Ministry of Education), Department of Genitourinary Oncology, Peking University Cancer Hospital & Institute, Beijing, China*

*^4^ Department of Orthopaed·ics, Guizhou Provincial People’s Hospital, Guiyang 550002, China*

*^#^ They contributed equally to this work.*

**Correspondence to: liuteli123321@163.com (T.L.), pekyz@163.com (Z.Y.), and zhuhuabch@pku.edu.cn (H.Z.)*

**This PDF file includes:**

I. Supplementary Methods

II. Supplementary Figure S1-5

III. Supplementary Table S1-3

IV. Supplementary Video

**Supplementary Methods**

**Synthesis of precursor DOTA-AP9**

The DOTA-AP9 peptide targeting CD147 was synthesized by standard solid-phase peptide synthesis on 2-chlorotrityl chloride resin. After resin swelling in N,N-Dimethylformamide (DMF), the first Fmoc-protected amino acid was coupled with N,N-Diisopropylethylamine (DIEA), followed by methanol capping and sequential Fmoc deprotection using 20% piperidine in DMF. Subsequent amino acids were successively coupled with HBTU/DIEA activation, and each coupling or deprotection step was verified by ninhydrin or Kaiser tests. After chain elongation, DOTA-tris(t-Bu ester) was conjugated to the N-terminus under the same coupling conditions. The resin was thoroughly washed and the peptide cleaved using a TFA/H_2_O/EDTA/TIS cocktail for 3 h, then precipitated with cold diethyl ether to obtain the crude product. The peptide was purified by preparative RP-HPLC (C_18_ column, 0.1% TFA in water/acetonitrile gradient), and fractions were analyzed by ESI-MS and analytical HPLC for molecular weight and purity (>95%). The purified DOTA-AP9 peptide was lyophilized and stored at −20 °C.

**CD147 expression analysis in different kinds of tumor cells**

The CD147 expression in A375, BXPC3, and A549 cells was determined by western blot (WB) and immunofluorescence (IF).

In the WB experiment, A375, BXPC3, and A549 cells were harvested. The total proteins were extracted from these cell lines and then subjected to BCA quantification (Beyotime Biotechnology Co, Beijing, China). Approximately 15 μg protein was loaded into each well of SDS-PAGE, followed by transferring onto nitrocellulose membranes. The membrane was blocked in 5% skimmed milk and probed with the primary antibody (Cat. 10186-RP01, Sino Biological, China) at 4 °C overnight. After incubation with HRP-conjugated secondary antibody (Cat. No. E-AB-1003, elabscience, China), CD147 protein expression was detected by enhanced chemiluminescence. The analysis of band densities was performed using Image J software (http://rsb.info.nih.gov/ij/, Bethesda, USA) with a loading control of GAPDH.

Furthermore, the expression of CD147 was measured by the IF staining. Cells were trypsinized, counted, and seeded into 24-well plates at a density of 5 × 10⁴ cells per well in 500 µL of medium. After incubation for 24 h at 37°C in a 5% CO₂ humidified incubator, the medium was removed, and cells were washed three times with precooled PBS. Fixation was carried out using 4% paraformaldehyde for 15 min at room temperature (RT), followed by three washes with 0.05% PBST. Cells were then blocked with 5% goat serum for 1 h at RT. Subsequently, cells were incubated with the primary antibody against CD147 overnight at 4°C. The next day, cells were washed and incubated with a fluorophore-conjugated secondary antibody (Cat. GB21303, Servicebio, China) for 1 h at RT in the dark. Nuclear staining was performed using DAPI for 10 min. Finally, slides were mounted with 90% glycerol and imaged using a fluorescence microscope equipped with a 63× oil immersion objective.

**Multiplex immunofluorescence staining**

Multiplex immunofluorescence (MxIF) was performed using the Opal 7‑Color Manual IHC Kit (Cat. RC0086Plus‑67RM; Recordbio Technology Co. Ltd., Shanghai, China) following the manufacturer’s protocol. Briefly, sections underwent antigen retrieval in EDTA buffer (pH 8.0) by microwave induction, endogenous peroxidase blocking with 0.3% hydrogen peroxide in methanol, PBST washing, and blocking with 5% bovine serum albumin (BSA) in PBS for 10 min. Primary antibodies included CD147 (Cat. 11989‑1‑AP, Proteintech, China), Ki67 (Cat. ab15580, Abcam, China), GLUT1 (Cat. 21829‑1‑AP, Proteintech, China), and MCT1 (Cat. 20139‑1‑AP, Proteintech, China), applied for 1 h, followed by a horseradish peroxidase–conjugated goat anti‑rabbit/mouse secondary antibody (Cat. RCB054; Recordbio) and fluorescent dye visualization. For multiplex staining, antibodies were stripped by repeating antigen retrieval before the next primary antibody incubation. Sections were counterstained with DAPI, mounted in glycerol–gelatin, and imaged on a DS‑U3 scanning microscope (Nikon). Confocal images (×20, scale bar = 50 μm) were analyzed for CD147⁺, Ki67⁺, MCT1⁺, and GLUT1⁺ areas using ImageJ.

**Immunohistochemistry (IHC) studies**

Tumor tissues were fixed in paraformaldehyde, paraffin‑embedded, and sectioned at 4–7 μm. Sections were deparaffinized, rehydrated, treated with 3% hydrogen peroxide at room temperature for 10 minutes to block endogenous peroxidase, and washed with PBS. Antigen retrieval was performed by microwave heating, followed by blocking with 0.5% goat serum for 50 minutes. Slides were incubated overnight at 4 °C with primary antibody (Cat. 11989‑1‑AP, Proteintech, China) and for 30 min with HRP‑conjugated goat anti‑rabbit IgG secondary antibody (Cat. GB2303, Servicebio, China). Immunoreactivity was visualized using a DAB substrate kit (Abcam, Boston, MA, UK) and counterstained with hematoxylin. Images were acquired using a Nikon/Olympus light microscope (E100/CX23, Japan) at ×20 magnification (scale bar = 50 μm).

**Supplementary Figures**


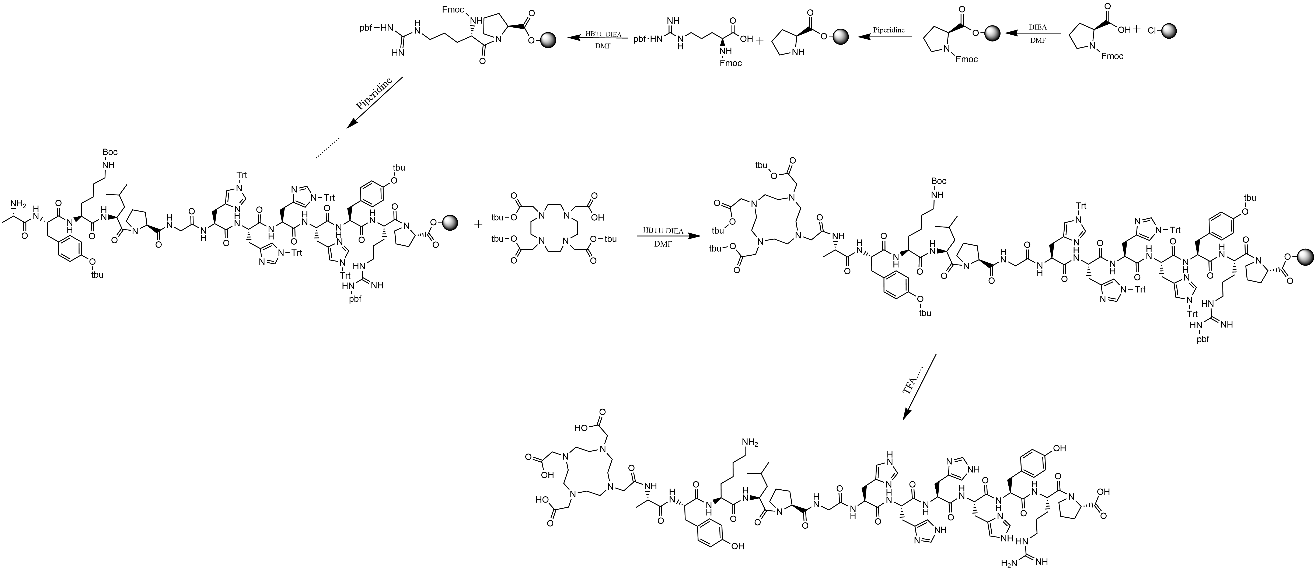


**Supplementary Figure 1. Synthetic route of peptide DOTA-AP9.**

**
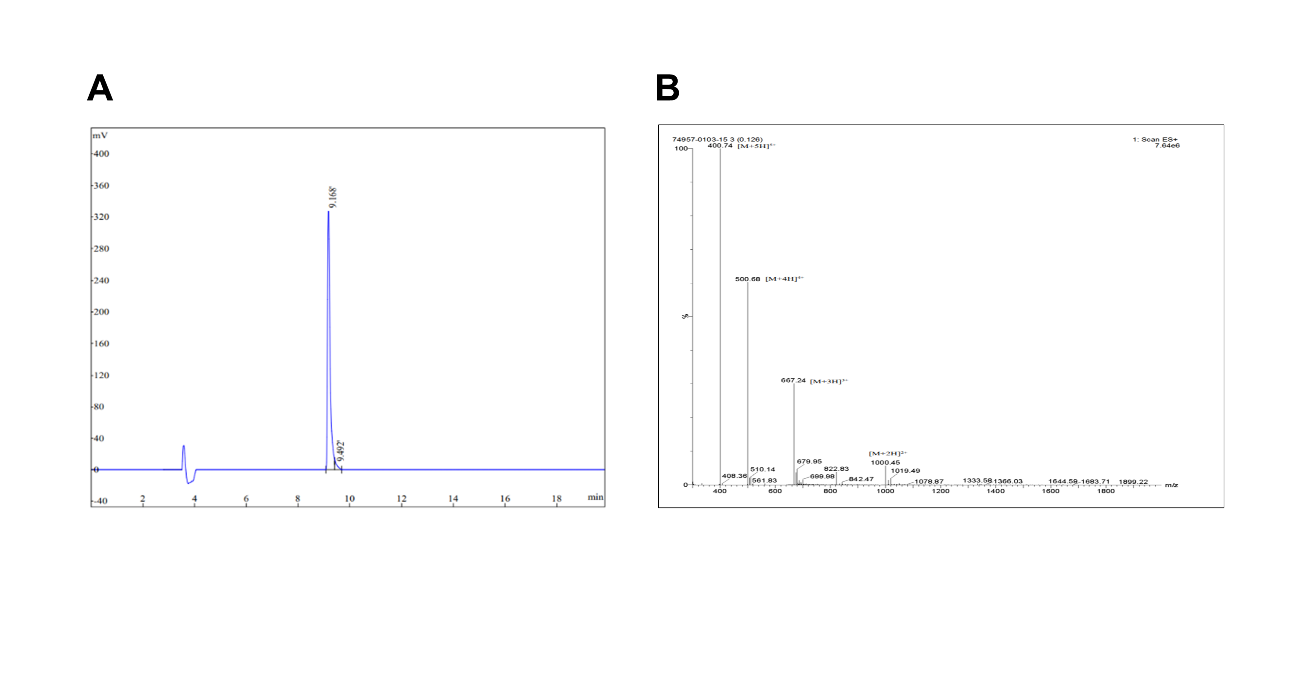
**

**Supplementary Figure 2. HPLC and ESI-MS analysis diagrams of the polypeptide DOTA-AP9.**

**
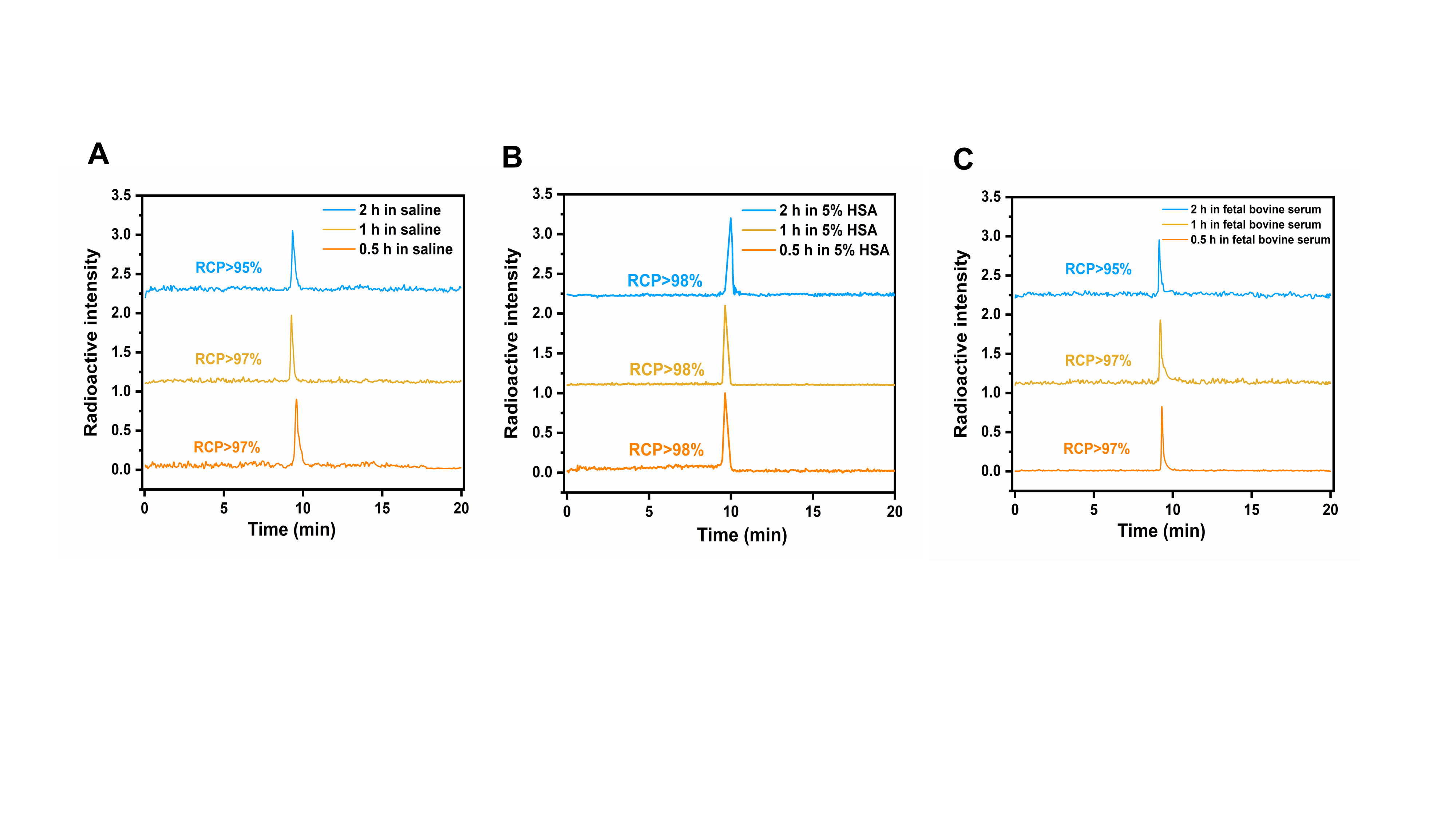
**

**Supplementary Figure 3. Radio-HPLC chromatograms of [^68^Ga]Ga-DOTA-AP9 incubated in saline, 5% HSA, and FBS for 0.5 h, 1 h, and 2 h.**

**
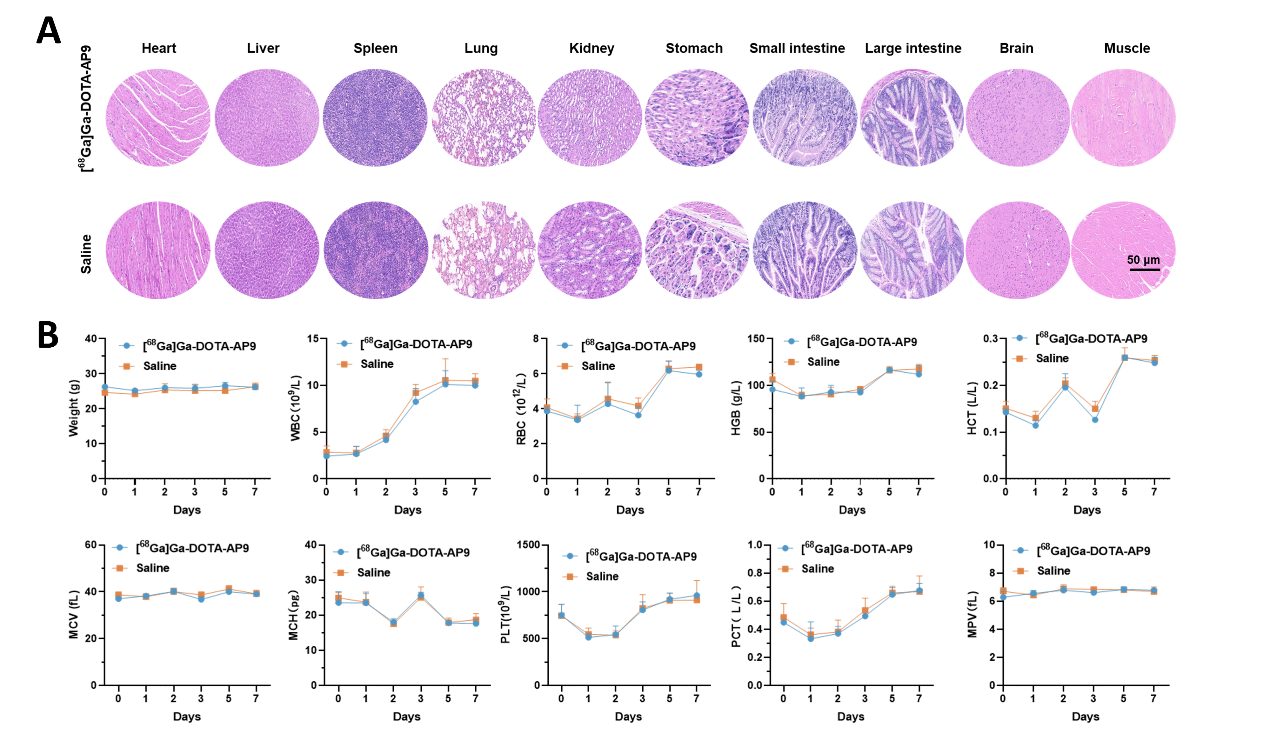
**

**Supplementary Figure 4. The results of body weight, blood routine detection and HE staining in the [^68^Ga]Ga-DOTA-AP9 probe experimental group and the normal saline control group.**

**
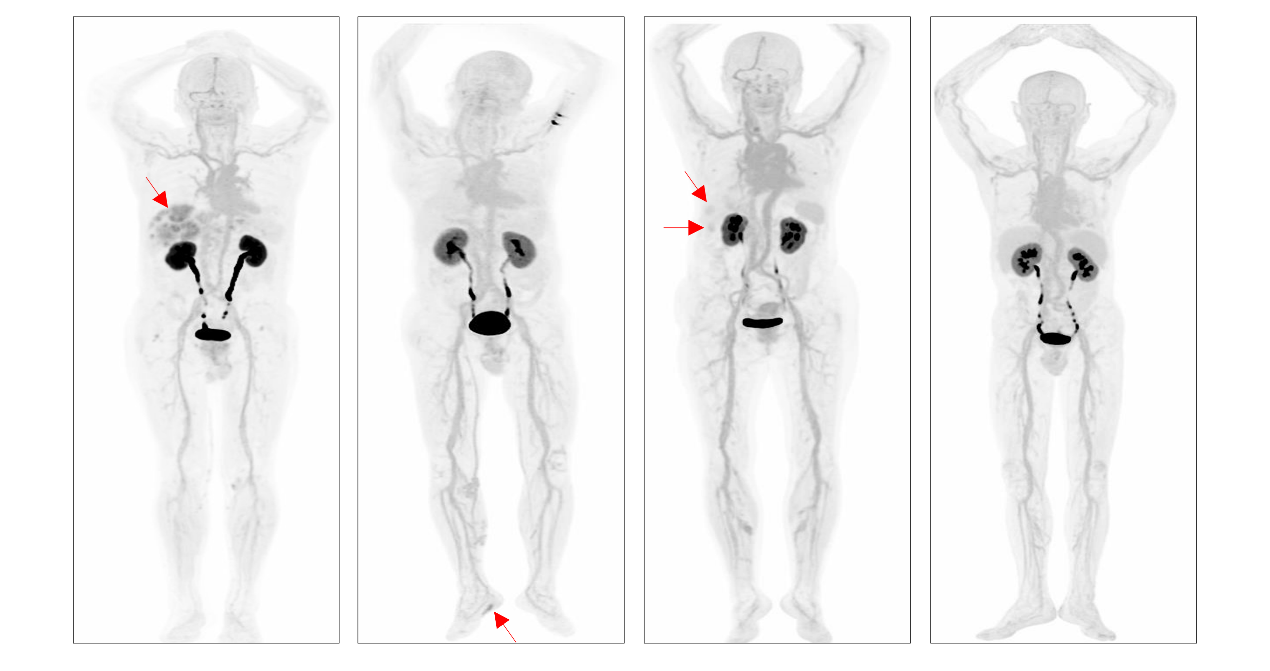
Supplementary Figure 5. Representative MIP images of [^68^Ga]Ga-DOTA-AP9 on participants with different CD147 expression levels at 30 min p.i.**

**Table S1 Biodistribution of [^68^**Ga]Ga-DOTA-AP9 probe in normal KM mice

| Organ | % ID/g (mean ± SD) | | | |
| --- | --- | --- | --- | --- |
|  | 5 min | 30 min | 1 h | 2 h |
| Blood | 5.15 ± 0.71 | 3.32 ± 0.09 | 0.94 ± 0.08 | 0.68 ± 0.11 |
| Heart | 1.52 ± 0.08 | 0.88 ± 0.09 | 0.61 ± 0.03 | 0.69± 0.13 |
| Liver | 1.88 ± 0.23 | 1.16 ± 0.07 | 1.26 ± 0.16 | 0.82 ± 0.08 |
| Spleen | 1.78± 0.20 | 1.45 ± 0.27 | 1.67 ± 0.31 | 1.32 ± 0.07 |
| Lung | 2.79 ± 0.80 | 1.13 ± 0.10 | 0.94 ± 0.03 | 0.77 ± 0.32 |
| Kindney | 16.75 ± 1.02 | 9.39 ± 1.64 | 7.68 ± 1.43 | 6.33 ± 0.81 |
| Stomach | 1.03 ± 0.07 | 0.71 ± 0.11 | 0.28 ± 0.03 | 0.51 ± 0.22 |
| Small intestine | 1.30 ± 0.13 | 0.71 ± 0.15 | 0.82 ± 0.11 | 1.01 ± 0.15 |
| Large intestine | 2.19 ± 0.44 | 1.32 ± 0.21 | 1.52 ± 0.40 | 1.16 ± 0.29 |
| Musle | 1.51 ± 0.03 | 0.69 ± 0.07 | 0.62 ± 0.09 | 0.49 ± 0.04 |
| Bone | 2.64 ± 0.36 | 2.17 ± 0.25 | 2.00 ± 0.24 | 1.65 ± 0.36 |
| Brain | 0.21 ± 0.02 | 0.11 ± 0.01 | 0.14 ± 0.02 | 0.14 ± 0.03 |

**Table S2 Estimation of the absorbed radiation dose and effective dose**

| Target organ | Absorbed Dose (mGy/MBq) |
| --- | --- |
| Adrenals | 2.12E-03 |
| Brain | 2.04E-03 |
| Breasts | 3.42E-04 |
| Esophagus | 7.57E-04 |
| Eyes | 7.59E-04 |
| Gallbladder Wall | 7.70E-04 |
| Left colon | 6.83E-04 |
| Small Intestine | 2.93E-03 |
| Stomach Wall | 6.78E-04 |
| Right colon | 5.51E-04 |
| Rectum | 4.15E-03 |
| Heart Wall | 1.37E-03 |
| Kidneys | 1.28E-02 |
| Liver | 3.73E-03 |
| Lungs | 2.97E-03 |
| Ovaries | 5.41E-04 |
| Pancreas | 8.40E-04 |
| Salivary Glands | 5.54E-04 |
| Red Marrow | 1.27E-03 |
| Osteogenic Cells | 1.79E-02 |
| Spleen | 5.19E-03 |
| Thymus | 6.43E-04 |
| Thyroid | 5.79E-04 |
| Urinary Bladder Wall | 3.90E-04 |
| Uterus | 5.37E-04 |
| Total Body | 2.03E-03 |
| Effective Dose（mSv/MBq） | 1.48E-03 |

**Table S3 The liver and renal function test results of [^68^**Ga]Ga-DOTA-AP9

| Type of test | Parameters | [^68^Ga]Ga-DOTA-AP9 | Control group |
| --- | --- | --- | --- |
| Liver function test | ALP | 103.78 ± 0.72 (U/L) | 109.15 ± 5.12 (U/L) |
|  | ALT | 32.15 ± 4.08 (U/L) | 37.78 ± 4 .65 (U/L) |
|  | AST | 134.55 ± 8.20 (U/L) | 131.63 ± 14.57 (U/L) |
| Renal function test | UREA | 12.64 ± 0.73 (mmol/L) | 13.24 ± 0.67 (mmol/L) |
|  | UA | 96.87 ± 5.61 (μmol/L) | 98.27 ± 7.87 (μmol/L) |
|  | CREA | 8.60 ± 0.22 (μmol/L) | 8.17 ± 0.40 (μmol/L) |
